# Severe Tetrodotoxin Poisoning after Consumption of *Lagocephalus sceleratus* (Pufferfish, Fugu) Fished in Mediterranean Sea, Treated with Cholinesterase Inhibitor

**DOI:** 10.1155/2012/782507

**Published:** 2012-10-01

**Authors:** Julia Kheifets, Boris Rozhavsky, Zehava Girsh Solomonovich, Rodman Marianna, Arie Soroksky

**Affiliations:** Intensive Care Unit, Wolfson Medical Center, Holon, Israel

## Abstract

*Lagocephalus sceleratus*, or better known as the pufferfish, or fugu, is widespread in Asia and Indo-Pacific regions. It is a poisonous fish containing tetrodotoxin (TTX) which is a potent neurotoxin. In the Far East, fugu is considered a delicate dish, especially in Japan where it is prepared by experts. Nevertheless, poisoning from *Lagocephalus sceleratus* is not a rare event. Recent data from Japan indicate an incidence of 45 patients per year and a mortality rate of 11%. Mediterranean sea is not the natural habitat of *Lagocephalus sceleratus*. However, by now multiple reports have established a firm presence of *Lagocephalus sceleratus* in Mediterranean region as well. This phenomenon is explained by migration of pufferfish across the Suez Channel (lessepsian migration) (Eisenman et al., 2008, Bentur et al., 2008). With lessepsian migration came the first reports of TTX poisoning in the Mediterranean region. We report a patient with a particularly severe and life-threatening TTX poisoning caused by consumption of *Lagocephalus sceleratus* and treated by cholinesterase inhibitor to a complete and uneventful recovery.

## 1. Case Report

**Figure 1:**
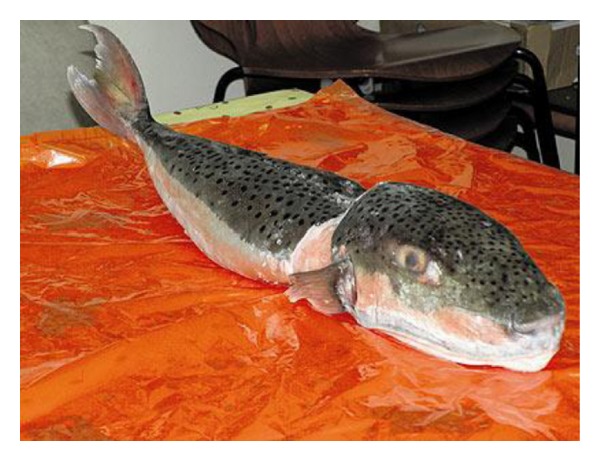
*Lagocephalus sceleratus* (Pufferfish, fugu) cought by a local recreational fisherman off the cost of Tel Aviv.

An otherwise healthy 52-year-old man presented to the emergency room with nausea and vomiting accompanied by acute dyspnea. Two hours earlier the patient who was a recreational fisherman reported on consumption of liver and gonads extracted from a fish which he just captured at sea (Figure 1).

Several minutes after consumption he complained of perioral paraesthesias with worsening limb muscle weakness. Shortly after admission to the ER, the patient developed an acute respiratory failure with bradypnea. This was accompanied by bradycardia which quickly deteriorated to cardiac arrest.

After a short resuscitation, including tracheal intubation and mechanical ventilation, the patient returned to sinus rhythm. On examination, shortly after patient was stabilized, signs of complete paralysis with absence of motor responses and lack of pupil reactions were noted. Patient was noted to be in deep coma with GCS of 3. At this point, possible poisoning by paralyzing agent was suspected. Patient was transferred to the intensive care unit and treated by supportive measures.

Several hours after his ICU admission, patient's family approached the medical stuff with a fish claimed to be consumed by the patient just two hours prior to his hospital admission. A diagnosis of tetrodotoxin (TTX) poisoning was suggested by typical clinical manifestations and temporal proximity to consumption of TTX-containing fish. The fish remnants were photographed and were immediately analyzed by a marine biologist and by the national center of poisoning. Consequently the fish was identified as the poisonous *Lagocephalus sceleratus*. At this point, the patient had a complete muscle paralysis with absent deep tendon reflexes and deep coma. Due to the severity of his illness, and after an extensive literature review, a decision was made to treat the patient with a cholinesterase inhibitor. During the first 24 hours the patient received 4 doses of intravenous neostigmine 2.5 mg. Immediately after the first dose of neostigmine deep tendon reflexes could be noted along with reversal of the comatose state.

Over the next 24 hours, the patient completed a course of 4 doses of neostigmine. During that time a dramatic improvement was observed, which included complete recovery of muscle strength and return to full consciousness. 36 hours after his hospital admission the patient was extubated and had a complete and uneventful recovery.

## 2. Discussion

Tetrodotoxin is a neurotoxin that occurs in select species of the family Tetraodontidae (pufferfish or fugu). It is an aminohydroxyquinazoline compound. In marine pufferfish species, toxicity is generally high in the liver and ovary, whereas in freshwater species, toxicity is higher in the skin. TTX is produced primarily by marine bacteria, and pufferfish accumulate TTX via the food chain that begins with these bacteria [1]. It is one of the most potent, nonprotein poisons known to man.

TTX blocks voltage-gated sodium channels and inhibits the production and propagation of action potentials, mainly in skeletal muscles, neurons, and nerve fibers. TTX-sensitive sodium channels exist also in heart muscle, and some signs of intoxication (as hypotension, conduction disorder, and bradycardia) are explainable by TTX action on these channels [1]. Symptoms of intoxication are urination, drooling, vomiting, diarrhea, absence of tendon reflexes, fasciculation, lethargy, ataxia, ascending progressive paralysis, and breathlessness.

Death is due to a rather complex action of TTX on the respiratory system, involving blockade of the phrenic nerve, the diaphragm, and neurons in the central respiratory network. TTX is rapidly eliminated from the serum not only by excretion in the kidney, but also via an efficient binding to the TTX receptors. Thus, the toxin may accumulate in an excitable tissue.

Low levels in the brain suggest a limited ability of TTX to cross the human blood-brain barrier [2]. In this context it is interesting to note that in mild cases of intoxication victims may remain conscious but completely paralyzed. However, coma has been reported in severe cases of TTX poisoning and in the final stages of intoxication before death.

The severity of poisoning depends on amount of toxin ingested. Usually TTX poisoning is diagnosed on basis of typical symptoms and anamnesis. Blood and urine samples can be analyzed using a TTX-specific enzyme-linked immunoassay (ELISA), and levels of tetrodotoxin correlate well with severity of symptoms [1]. Intoxication can be rapidly fatal, and antidotes do not exist. The human killing dose is assumed to be 1-2 mg. Treatment is supportive and may involve mechanical ventilation, use of intravenous fluids and vasopressors; cholinesterase inhibitors have been suggested but not tested adequately [3]. Prognosis is good if the patient arrives at the emergency department conscious and prior to respiratory arrest, and if the patient survives the first 24 hours. Thereafter, symptoms usually resolve over a period of 24 hours to five days [1].

Tetrodotoxin poisoning is quite common in Japan and South-East Asia (secondary to consuming of meals prepared from puffer fish or “fugu” fish). Preparation of these gourmet foods is strictly regulated in Japan, and most of intoxication cases now are related to illegal or home-prepared fish. Differently, in other regions TTX intoxication is mostly related to accidental consumption of pufferfish by uninformed eaters. A report from 2008 of 141 patients with pufferfish poisoning in Bangladesh found mortality rate of 12%, mostly due to respiratory failure caused by severe muscle weakness or paralysis [3].

Since 2007 one species of pufferfish (*Lagocephalus sceleratus*) was repeatedly reported as the cause of TTX poisoning in Mediterranean region (Israel, Lebanon) [4]. 13 cases of TTX poisoning were registered by Israel Poison Information Center in December 2008; all cases were related to consumption of poisonous fish *Lagocephalus sceleratus* [4]. Symptoms of poisoning included perioral paraesthesia, tingling over the entire body, nausea and vomiting, dizziness, headache, abdominal pain, and muscular paralysis of the limbs.

Intensive supporting measures to ensure adequate oxygenation and hemodynamic stabilization are life saving. The use of neostigmine has been reported sporadically [2]. TTX toxicity is caused by competitive and reversible block at the motor end plate as well as motor axon and muscle membrane. Increasing the release of acetylcholine at the neuromuscular junction by anticholinesterase drugs may reverse this block. Thus, neostigmine, which reversibly binds and inactivates acetyl cholinesterase, can increase acetylcholine availability at the neuromuscular junction and dramatically restore transmission. It also acts on the central and the peripheral nervous systems, the autonomic motor, and sensory nerves.

Our patient presented with a severe muscle weakness that quickly deteriorated to muscle paralysis. This paralysis was reversed immediately after administration of neostigmine. With repeated doses (up to 4 doses), muscle strength continued to improve dramatically until complete and uneventful recovery.

This case illustrates the need for high index of suspicion for TTX intoxication, especially when presented with a clinical picture of muscle paralysis in the context of consumption of suspicious fish and the potential beneficial effects of treatment with cholinesterase inhibitors such as neostigmine for rapid reversal of TTX toxicity.
